# COMPARATIVE EVALUATION OF LONG PULSE ALEXANDRITE LASER AND INTENSE PULSED LIGHT SYSTEMS FOR PSEUDOFOLLICULITIS BARBAE TREATMENT WITH ONE YEAR OF FOLLOW UP

**DOI:** 10.4103/0019-5154.57615

**Published:** 2009

**Authors:** Tahra M Leheta

**Affiliations:** *From the Department of Dermatology, Cairo University, Cairo, Egypt.*

**Keywords:** *Intense pulsed light*, *long-pulse Alexandrite laser*, *pseudofolliculitis barbae*

## Abstract

**Background:**

Existing remedies for controlling pseudofolliculitis barbae (PFB) are sometimes helpful; however the positive effects are often short lived. The only definitive cure for PFB is permanent removal of the hair follicle.

**Aims:**

Our aim was to compare the efficacy of the Alexandrite laser with the intense pulsed light system in the treatment of PFB and to follow up the recurrence.

**Methods:**

Twenty male patients seeking laser hair removal for the treatment of PFB were enrolled in this study. One half of the face was treated with the long-pulse Alexandrite laser and the other half was treated with the IPL system randomly. The treatment outcome and any complications were observed and followed up for one year.

**Results:**

All patients exhibited a statistically significant decrease in the numbers of papules. Our results showed that the Alexandrite-treated side needed seven sessions to reach about 80% improvement, while the IPL-treated side needed 10-12 sessions to reach about 50% improvement. During the one year follow up period, the Alexandrite-treated side showed recurrence in very minimal areas, while the IPL-treated side showed recurrence in bigger areas.

**Conclusions:**

Our results showed that both systems might improve PFB but Alexandrite laser was more effective at reducing PFB than IPL.

## Introduction

Pseudofolliculitis barbae (PFB) is an inflammatory condition of the beard area usually observed in men with thick coarse hair. Typically, the follicle is at an acute angle to the skin surface, and the sharpened end of the shaved shaft reenters the skin at or next to the follicular opening. The subsequent formation of inflammatory papules and pustules can result in patient discomfort, secondary infection, and even hypertrophic or keloidal scars.[1,2] Growing a beard is usually curative, at least in the early stages of the disease.[3]

Existing remedies for controlling PFB include topical steroids, antibiotics, and exfoliating agents. Although these agents are sometimes helpful in the treatment and management of PFB, the positive effects are often short lived. Physical modalities such as electrolysis have also been used in the treatment, but this technique, in addition to being tedious, can cause pigmentation abnormalities, scarring, and residual keratin abscesses from fragmentary destruction of the hair follicle.[4–7] For some patients with PFB, the best treatment is maintaining a neatly trimmed “beard” with an average “above the surface” hair length of about 1/8 inch.[3]

The only definitive cure for PFB is permanent removal of the hair follicle. This serves as the basis for the use of newer surgical modalities. Laser hair removal with a variety of laser systems [diode (800-810 nm), pulsed Alexandrite (755 nm), Neodymium:Yttrium Aluminum Garnet (Nd:YAG (1064 nm), and pulsed non coherent light source have been used.[8] Hair follicles are targeted for removal with melanin acting as the chromophore. Because melanin is targeted with this procedure, side effects predictably occur most frequently in dark skinned patients. Adverse effects include epidermal changes and pigmentary alterations. These side effects can be reduced by selecting a pulse duration that is shorter than that of the thermal relaxation time of the hair follicle and by using skin cooling techniques. Other side effects of laser hair removal include erythema, crusting, blistering, and scarring. Proper laser technique (minimal overlap and appropriate skin cooling) can significantly minimize these effects.[9,10].

Our aim is to compare the efficacy of the Alexandrite laser with the intense pulsed light system in the treatment of PFB and to follow up the recurrence.

## Materials and Methods

### Subjects

A total of 20 male patients seeking laser hair removal for the treatment of PFB were enrolled in this study. The protocol was approved by the Dermatology Research Ethics Committee Office, Faculty of Medicine, Cairo University. Fifteen patients completed the study. Of the five patients who were unable to complete the study, two patients failed to attend for part of their treatment at the correct time and were excluded from the study. In addition, one patient was excluded as he developed skin hypersensitivity to laser treatment resulting in significant blistering even at low fluences, and the other two lost interest. Of the 15 patients completing the study, the mean age was 28.8 years (average 18-43). All patients had skin phototypes II to IV, with coarse curly hair ranging in color from brown to black. Each patient had a history of PFB in the treatment area for a minimum duration of 1 year. Previous failed treatments included topical antibiotics, oral antibiotics, and intralesional corticosteroid injections. Exclusion criteria included a history of keloidal scarring, active herpes simplex in the treatment area, isotretinoin use in the past 6 months, pregnancy, vitiligo, recent laser therapy, or electrolysis in the study area.

### Study protocol

This study was a randomized, double blinded, split face controlled trial comparing the GentleLase Alexandrite laser (Candela Corp., Wayland, MA) with the Chromolite intense pulsed light system (Chromogenex technologies Ltd., LLanelli, UK). The set up and fluences used for the two systems are described below. In both cases the fluences used were within the recommended range by the manufacturers and those commonly used for the purposes of hair removal. The study aimed to directly compare these two systems by treating one side of the face using the Alexandrite laser and the other side with the IPL. Envelopes were made up randomizing IPL treatment to either right or left and Alexandrite laser treatment to the opposite side. The envelopes were opened immediately prior to the first treatment. Response to treatment on the two sides of the face was finally assessed 12 months after the start of the treatment and 1 year after treatment cessation to evaluate the recurrence.

### Alexandrite laser

The GentleLase Alexandrite laser used in this study has a wavelength of 755 nm and 3 milliseconds pulse duration. All patients were treated using a 15 mm spot and accompanying dynamic cooling device. Standard starting fluences of 10 J/cm^2^ were used, with fluences subsequently increased to between 16 and 18 J/cm^2^ as tolerated.

### Intense pulsed light system

The chromolite intense pulsed light system employed in the study incorporated a 610-1200 nm filter on the flash lamp with selectable pre set programs for direct energy control and Smarlite^®^ technology. Treatments were carried out using a spot size of 50 × 15 mm. Epidermal cooling was achieved using a thin layer of cooled ECG gel. Patients were treated using 12-15 J/cm^2^.

### Interventions

Patients were instructed not to shave for 2 weeks before treatment (to allow decreased inflammation at the sites). They shaved on the morning of therapy, after which they resumed shaving and clipping (whatever their normal protocol was) beginning first week after treatment.

After informed consent was obtained, the affected areas were treated with contiguous pulses, and care was taken to stretch the skin during treatment to ensure close contact between the hand piece and the patient's skin. After treatment, patients were instructed to only apply a topical antiseptic and topical steroid for 24 hours.

Follow up visits and additional treatments for one year were performed at 4 to 6 week intervals for four sessions, then at 4 to 8 week intervals. During each visit, photographs were taken, and an additional treatment was performed as before. PFB lesion and hair counts in a predefined fixed area were made by a blinded observer. Shaving bumps were counted on each half of the neck in a side by side comparison. Grading was performed comparing pretreatment and posttreatment photographs. A quartile grading system was used to rate papule formation and hair density reductions as follows: 1 indicated 0% to 25% improvement; 2-26% to 50%; 3-51% to 75%; and 4-greater than 75%.

## Results

Fifteen male patients with PFB completed this study. All patients tolerated the laser treatments well and experienced minimal discomfort. Perifollicular edema and erythema developed immediately after treatment and lasted 1 to 2 days. Blister formation did not occur in any of the treatment areas. Two patients reported scattered crusts after the first treatment, at the IPL treated side that resolved in 2 to 3 days.

All patients were uniformly satisfied with their treatment and noted improvement after just 1 treatment session.

**Figure 1:**
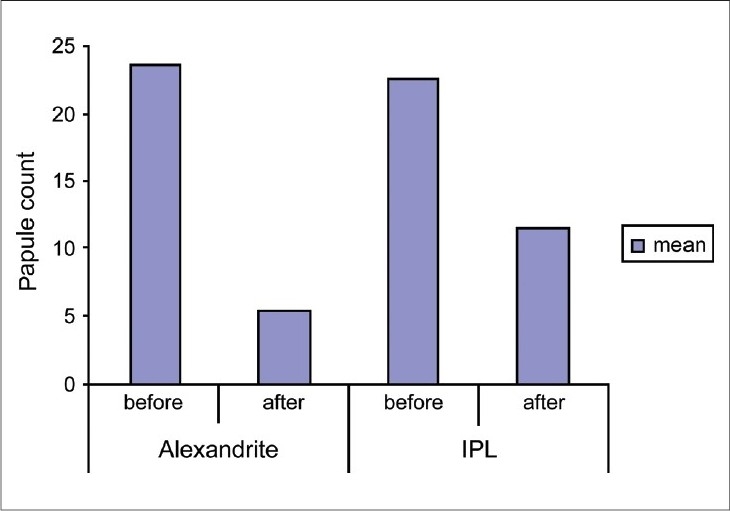
The mean of PFB lesion count before and after treatment

After treatment, all subjects improved; The Alexandrite-treated side baseline mean count was 22.8 ± 8.562 SD and at the end of the study showed a mean of 4.733 ± 1.751 SD (*P* < 0.001), and the IPL-treated side showed a mean baseline PFB lesion count of 21.8 ± 8.169 SD, which decreased to a mean of 10.8 ± 4.074 SD (*P* < 0.001) as determined by a 2 tailed paired *t* test [Figures 1–3].

Our results showed that the Alexandrite-treated side needed seven sessions to reach about 80% improvement (grade 4), while the IPL-treated side needed 10-12 sessions to reach about 50% improvement (grade 2) [Figures 2 and 3].

**Figure 2a:**
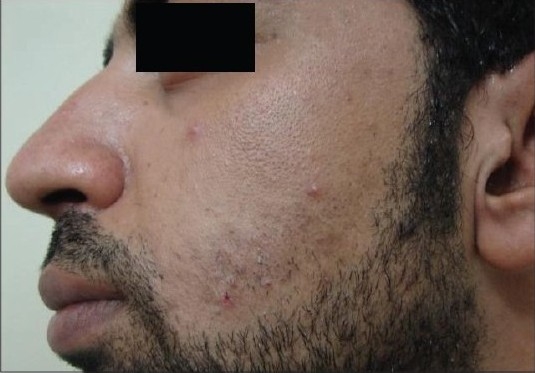
Patient 1 with the IPL-treated side before treatment

**Figure 2b:**
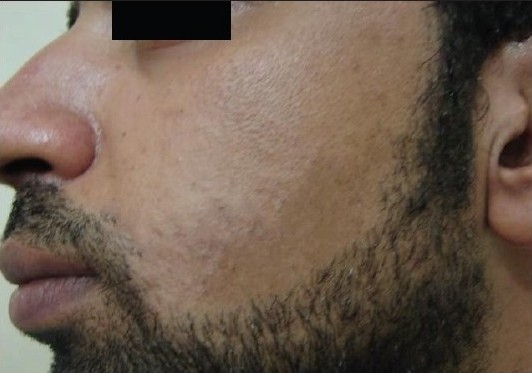
Patient 1 with the IPL-treated side after treatment

**Figure 2c:**
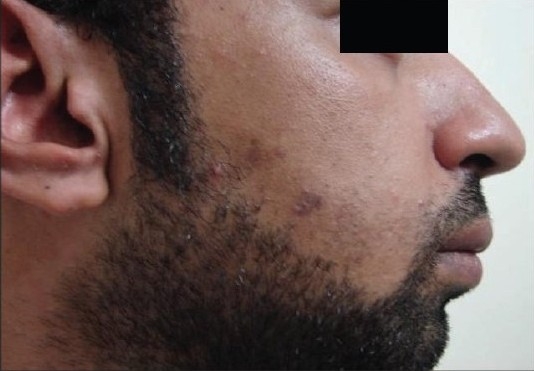
Patient 1 with the Alexandrite-treated side before treatment

**Figure 2d:**
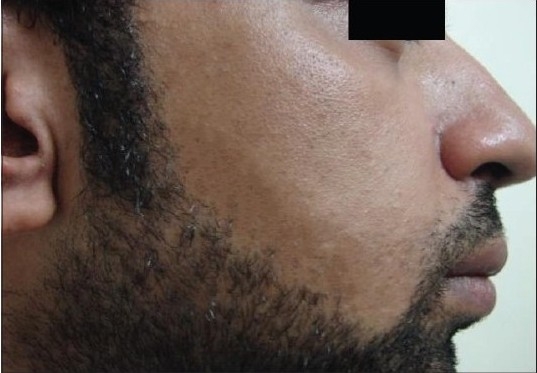
Patient 1 with the Alexandrite-treated side after treatment

**Figure 3a:**
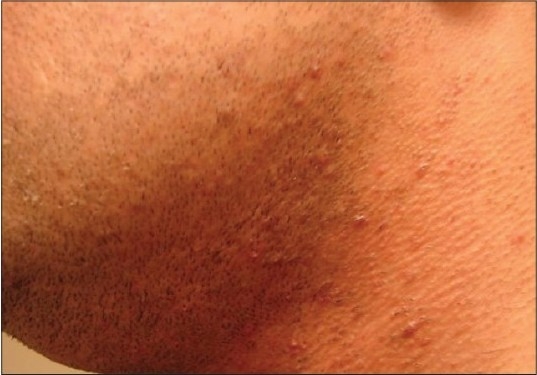
Patient 2 with the Alexandrite-treated side before treatment

**Figure 3b:**
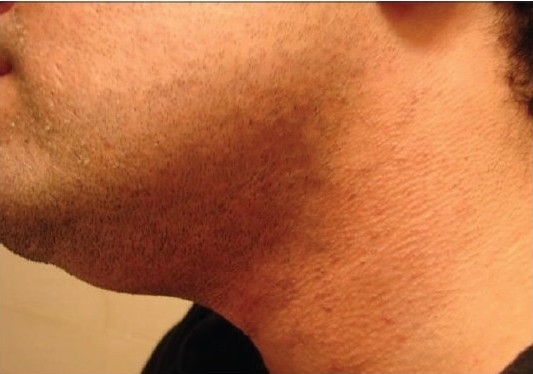
Patient 2 with the Alexandrite-treated side after treatment

**Figure 3c:**
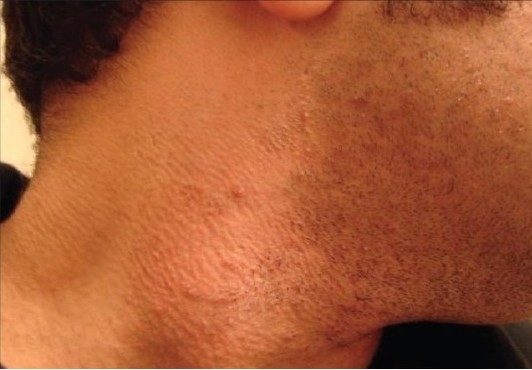
Patient 2 with the IPL-treated side before treatment

**Figure 3d:**
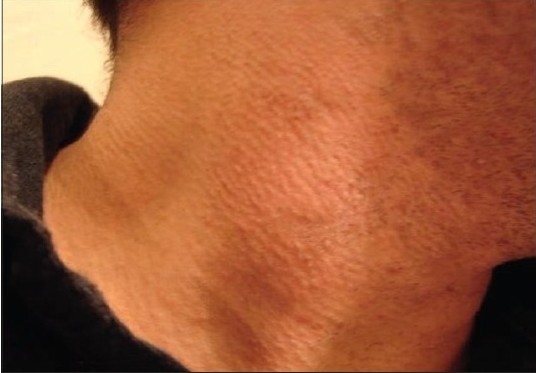
Patient 2 with the IPL-treated side after treatment

During the one year follow up period, the Alexandrite-treated side showed recurrence in very minimal areas which needed a session every 6 months, while the IPL-treated side showed recurrence in bigger areas which needed a session every 3 months.

## Discussion

Pseudofolliculitis barbae is a common disorder of glabrous skin in individuals with coarse, curly hair that develops following hair removal, most commonly by shaving. Other hair removal methods, including wax epilation, plucking, and electrolysis, also can result in pseudofolliculitis barbae. Traditional therapies, including topical and oral antibiotics, tretinoin cream, corticosteroids, and laborious shaving methods, have yielded unsatisfying results. The most obvious treatment for this disorder is complete follicular destruction. Laser hair removal is therefore a logical therapeutic approach to this common and difficult problem.[9]

To reduce hair density with lasers, the principle of selective photothermolysis is usually employed. Light-based devices are designed to heat the hair follicle selectively while sparing epidermal melanin and the perifollicular dermis. Melanin absorption decreases with longer wavelengths. In addition, because scattering decreases as a function of wavelength,[11] longer wavelengths penetrate deeper into the dermis. At wavelengths close to 700 to 800 nm, melanin absorption is about three to five times that at 1,064 nm. Much of it (compared with 1064 nm) is absorbed by the epidermis, so in darker skin, there is an increased risk of epidermal damage.[12] To achieve extreme localized heating, pulse durations that are smaller than the thermal relaxation time (t) of the hair follicles (between 10 and 100 ms) have traditionally been used.[13–15] For shorter wavelengths (700-800 nm), epidermal damage can be minimized by selecting pulse durations that are longer than the thermal relaxation time of the epidermis, which is in the range of 3 to 10 ms. By lengthening the pulse duration to much greater than thermal relaxation time of the epidermis, even greater epidermal sparing can be achieved.[16]

Ross *et al.,*[3] previously stated that the elimination and miniaturization of hair shafts was positively correlated with a decrease in the number of inflammed papules characteristic of PFB. They suggested, not unexpectedly, that the hair shaft itself was the principal contributor to inflammation and pustulation in patients with PFB.

Using this strategy for PFB, delaying hair growth was the goal of our study as delaying hair growth reduced the frequency and vigor of shaving and therefore might reduce the severity of the disease.

This study has demonstrated that treatment with the Alexandrite laser and the IPL improved PFB. All patients exhibited a statistically significant decrease in the number of papules. This study showed that treatment with the Alexandrite-treated side needed about seven sessions to improve PFB by about 80%, while the IPL-treated side showed about 50% improvement in 10-12 sessions.

Previous investigators have used lasers in the treatment of PFB. Nanni *et al.*,[17] reported the use of a long pulsed Alexandrite laser, but noted that the hair reduction was temporary. They also suggested that part of the decrease in papules and pustules might be because of a gentle exfoliative effect often observed after treatment. Rogers and Glaser[18] reported that the effective use of a Q switched Nd:YAG laser and topical carbon suspension leads to the reduction of inflammatory papules and pustules. Chui *et al*.,[19] used a normal mode ruby laser in a white patient with PFB and observed improvement even 10 months after the last of 3 treatments. Battle *et al*.,[20] have reported the use of a novel long-pulsed 800-nm laser (20-200 ms) in darker skin types (up to type VI). They achieved safe and effective hair reduction by combining lower fluences with longer pulses. Kauvar[9] has showed reduction in the severity and number of shaving bumps with a more traditional 800 nm. The rationale for this treatment was to eliminate the cause of PFB by reducing the number of coarse hairs in the affected areas.

Questions remained regarding this strategy for treating PFB. One issue was the permanence of the hair loss. Extended observations have shown that during the follow up period of the patients for 1 year; there was clinically apparent hair reduction and PFB disappearance at the treated sites. However, we have noted that some hairs have grown again in very minimal areas at the Alexandrite-treated side, while at the IPL-treated side hairs have grown again in bigger areas. However, these hairs were thinner than their pretreatment diameters and needed fewer sessions (3 to 6 monthly treatments) to show long term improvement.

It was clear from the results in this study that the GentleLase Alexandrite laser was more effective at reducing PFB than the Chromolite IPL. It was probable that this was due to the specific wavelength, short pulse duration and single pulse delivery of the GentleLase Alexandrite laser, resulting in more follicular destruction than the IPL where the energy delivered was split. Despite the poorer results, the chromolite IPL did still result in a significant reduction in hair growth and PFB improvement and patients were satisfied with treatment. As the IPL can be used to treat a wide variety of other conditions simply by changing the filter used, and is significantly cheaper to purchase than the Alexandrite laser, it still has a role to play in the treatment of PFB, particularly where one system is wanted to treat a variety of different conditions.

## References

[1] Alexander AM, Delph WI (1974). Pseudofolliculitis barbae in the military: A medical, administrative and social problem. J Natl Med Assoc.

[2] Ross EV, Chhieng N (1999). Lasers in the military for cutaneous disease and wound healing. Dermatol Clin.

[3] Ross EV, Cooke LM, Timko AL, Overstreet KA, Graham BS, Barnette DJ (2002). Treatment of pseudofolliculitis barbae in skin types IV, V, and VI with a long-pulsed neodymium:Yttrium aluminum garnet laser. J Am Acad Dermatol.

[4] Braunder GJ, Flandermeyer KL (1977). Pseudofolliculitis barbae. 2. Treatment. Int J Dermatol.

[5] Brown LA (1983). Pathogenesis and treatment of pseudofolliculitis barbae. Cutis.

[6] Halder RM (1988). Pseudofolliculitis barbae and related disorders. Dermatol Clin.

[7] Galaznik JG (1984). A pseudofolliculitis barbae clinic for the black male who has to shave. J Am Coll Health.

[8] Perry PK, Cook-Bolden FE, Rahman Z, Jones E, Taylor SC (2002). Defining pseudofolliculitis barbae in 2001: A review of the literature and current trends. J Am Acad Dermatol.

[9] Kauvar AN (2000). Treatment of pseudofolliculitis with a pulsed infrared laser. Arch Dermatol.

[10] Haas AF (2000). Use of a unique cooling gel applied prior to laser hair removal. Dermatol Surg.

[11] Jacques SL (1992). Laser-tissue interactions. Photochemical, photothermal, and photomechanical. Surg Clin North Am.

[12] Handrick C, Alster TS (2001). Comparison of long-pulsed diode and longpulsed Alexandrite lasers for hair removal: A long-term clinical and histologic study. Dermatol Surg.

[13] Campos VB, Dierickx CC, Farinelli WA, Lin TY, Manuskiatti W, Anderson RR (2000). Ruby laser hair removal: Evaluation of long-term efficacy and side effects. Lasers Surg Med.

[14] Dierickx C, Alora MB, Dover JS (1999). A clinical overview of hair removal using lasers and light sources. Dermatol Clin.

[15] Grossman MC, Dierickx C, Farinelli W, Flotte T, Anderson RR (1996). Damage to hair follicles by normal-mode ruby laser pulses. J Am Acad Dermatol.

[16] Altshuler GB, Anderson RR, Manstein D, Zenzie HH, Smirnov MZ (2001). Extended theory of selective photothermolysis. Lasers Surg Med.

[17] Nanni C, Brancaccio R, Cooperman M (1999). Successful treatment of pseudofolliculitis barbae with a long pulsed Alexandrite laser. Lasers Surg Med Suppl.

[18] Rogers CJ, Glaser DA (2000). Treatment of pseudofolliculitis barbae using the Q-switched Nd:YAG laser with topical carbon suspension. Dermatol Surg.

[19] Chui CT, Berger TG, Price VH, Zachary CB (1999). Recalcitrant scarring follicular disorders treated by laser-assisted hair removal: A preliminary report. Dermatol Surg.

[20] Battle E, Suthamjariya K, Alora M, Palli K, Anderson R (2000). Very long pulses (20-200 ms) diode laser for hair removal on all skin types [abstract]. Lasers Surg Med Suppl.

